# Where There Is No Health Research: What Can Be Done to Fill the Global Gaps in Health Research?

**DOI:** 10.1371/journal.pmed.1001209

**Published:** 2012-04-24

**Authors:** Martin McKee, David Stuckler, Sanjay Basu

**Affiliations:** 1 European Centre on Health of Societies in Transition, London School of Hygiene & Tropical Medicine, London, United Kingdom; 2 Department of Sociology, Cambridge University, Cambridge, United Kingdom; 3 Department of Medicine, University of California San Francisco, San Francisco, California, United States of America; 4 Division of General Internal Medicine, San Francisco General Hospital, San Francisco, California, United States of America

## Abstract

As part of a cluster of articles leading up to the 2012 World Health Report and critically reflecting on the theme of “no health without research," Martin McKee and colleagues examine the question of what to do to build capacity in the many countries around the world where health research is virtually non-existent.

**Summary Points**

- Efforts to strengthen capacity in health research have, so far, concentrated on countries where there is existing capacity rather than those where it is almost completely lacking.
- Judged by absolute numbers of scientific papers, those with the fewest are mainly small islands and a few countries that are politically isolated.
- Judged by papers per capita, the lowest include countries in the former Soviet Union and Africa, both regions experiencing declines in life expectancy in recent years, and states experiencing conflict.
- Although there is a positive association between economic development and research output, some relatively wealthy countries seriously underperform.
- There are many examples of good practice, including regional networks and international partnerships.
- There is a strong argument for donors to look to the long term and consider how best to build health research capacity where it is virtually absent.

*In anticipation of the 2012 *World Health Report*, this paper was commissioned to help contextualize and critically reflect on the theme of “no health without research."*

## Introduction

Forty years ago Archie Cochrane, in his seminal book *Effectiveness and Efficiency*, drew attention to the concentration of health research, and particularly clinical trials, in a very few high-income countries [1].The situation has changed remarkably since, but there are still many countries where health research is virtually non-existent. These countries have, so far, received almost no attention in the international literature on health research and are overlooked by funders, who feel that they can only invest where there is sufficient existing capacity to absorb resources. We ask what might be done to help these countries.

Clearly, a first step is to make a case for the importance of establishing national health research strategies. Such programmes are essential for public health systems to function and thrive. Without answers to core questions, such as “who is most in need?" and “what do they suffer from?", it is impossible to determine “which resources would help people recover?" It is crucial that the scope be “national"; missing data are frequently imputed from neighbouring countries even though conditions may be quite different. Imputed data also fail to capture the distribution of health and its determinants within countries. Furthermore, even if national public health authorities are able to conduct limited surveillance among parts of their populations, without a health research strategy they are unlikely to be able to capture and prepare for changes in their citizenry's health, whether it be a new infectious disease or the more gradual development of a “dual burden" of infectious and non-communicable diseases. Finally, without research, they cannot know whether what they are doing is actually working.

## Which Countries Face the Greatest Health Research Deficits?

There is no comparable international indicator of how much health research various populations have access to. One crude measure of capacity is the output of medical research publications by researchers based in institutions in each country (i.e., not research on populations by researchers based in other countries). We have used the SCImago database, which is based on the SCOPUS database [2], one of a number of bibliometric databases. Although there is no gold standard database for tracking publications [3], SCImago has several important advantages for our purposes as it covers more journals than Web of Science (about 15,000) and provides better coverage of publications in languages other than English than do its competitors and, should others wish to extend our analyses, its method of calculating citation factors is also more inclusive than other databases [4]. Finally, it is also open-access, making our calculations easy to update and highly replicable [5]. Although the per capita output of publications is a crude indicator of research capacity, it is the only source of reasonably comparable global data to indicate how much health research is undertaken by whom. Other measures, such as numbers of researchers, are fraught with definitional problems [6]. Yet, although number of publications is the best measure available, it is important to recognise its limitations. It does not capture quality of publications (if such a measure exists) [7], although there is a relatively close correlation between the number of publications per capita from each country and the average h-index, a measure of both scientific productivity and frequency of citations of researchers in each country (*r* = 0.61, authors' calculations). Nor does it distinguish the individuals being researched, such that substantial within-country inequalities in access to research may be masked.

Table 1 shows the 25 countries or territories with the fewest number of indigenous publications in the field of medicine over the past 15 years. Unsurprisingly, almost all of the countries are sparsely populated small islands. The three with fewest published health research studies (Cocos [Keeling] Islands, Christmas Island, and Tokelau) each have fewer than 2,000 inhabitants. There are, however, two notable exceptions, both of which owe their position to their self-imposed isolation from the global community. The Democratic Republic of Korea (North Korea) has produced only five publications, despite being a nuclear power. Turkmenistan, which since independence has also been under a near-dictatorship, is not quite as isolated commercially, given its large-scale production of natural gas, but its academic medical community faces formidable barriers in engaging with the rest of the world. In 2004, the former president fired 15,000 public health workers and, the following year, closed all hospitals outside the capital as well as all libraries, reasoning that any knowledge his citizens required could be found either in the Koran or a book of his own writings, the Ruhnama. Health statistics are believed to be routinely falsified, and access to the Internet is extremely limited [8].

**Table 1:** Countries and territories with the fewest publications in medicine (1996–2010) in absolute numbers.

Rank	Country	Publications	Publications/100,000 Population
200	Mayotte	15	7.35
201	Timor-Leste	15	1.33
203	Palau	14	68.39
202	Comoros	14	1.91
205	Marshall Islands	13	24.06
204	Aruba	13	12.09
206	Virgin Islands (British)	11	40.74
208	Northern Mariana Islands	9	14.77
207	Cape Verde	9	1.81
209	Turkmenistan	8	0.16
210	Norfolk Island	7	324.83
213	United States Minor Outlying Islands	6	1904.76
211	Gibraltar	6	20.52
214	Saint Vincent and The Grenadines	6	5.49
212	Saint Lucia	6	3.45
215	Democratic Republic of Korea	5	0.02
216	Cook Islands	4	32.60
217	Kiribati	3	3.01
219	Falkland Islands (Malvinas)	2	67.68
220	Tuvalu	2	20.35
218	Anguilla	2	14.81
221	Wallis and Futuna	2	13.33
222	Cocos (Keeling) Islands	1	166.67
223	Christmas Island	1	71.28
224	Tokelau	1	70.87

Note: The term “United States Minor Outlying Islands" encompasses a group of Pacific atolls with no permanent population. While featured in only six publications, it has a high proportion of scientists among the 300 or so temporary visitors, incidentally, making it the territory with the highest number of publications per head of population in the world.

It is important to look at publications in relation to population size (Table 2). Myanmar is now included in this list. Given Myanmar's traditionally strong higher education sector, this suggests that political isolation is playing a role. Many of the countries on this list of research deprivation fall into two broad geographical groupings: African countries and countries of the former Soviet Union. These two regions stand out for being the only two in which mortality has risen over the past several decades [9]. Others are countries with histories of significant conflict: Yemen, Timor-Leste, and Afghanistan. The poor performance of Indonesia has recently been noted by the country's director general of health research, although the proposed remedy, involving a focus on increasing publications by students, rather than tackling more fundamental weaknesses, has been controversial [10].

**Table 2:** Countries and territories with the fewest publications in medicine (1996–2010) per capita.

Rank	Country	Publications	Publications/100,000 Population
200	Cape Verde	9	1.81
201	Kyrgyzstan	92	1.71
203	Mozambique	399	1.71
202	Niger	255	1.64
205	Mauritania	54	1.56
204	Yemen	367	1.53
206	Ethiopia	1,265	1.53
208	Sierra Leone	86	1.47
207	Kazakhstan	239	1.46
209	Timor-Leste	15	1.33
210	Guinea	113	1.13
213	Eritrea	57	1.08
211	Indonesia	1,948	0.81
214	Burundi	66	0.79
212	Uzbekistan	201	0.71
215	Liberia	27	0.68
216	Chad	51	0.45
217	Angola	84	0.44
219	Afghanistan	145	0.42
220	Tajikistan	29	0.42
218	Myanmar	192	0.40
221	Somalia	21	0.23
222	Turkmenistan	8	0.16
223	Democratic Republic Congo	87	0.13
224	Democratic Republic of Korea	5	0.02

It is also important to consider availability of resources. For those countries for which data are available (i.e., other than small island states, a few states engaged in ongoing conflict, and dependent territories), there is a close correlation between publications and gross national product (GNP) per capita (*r* = 0.723). However, there is an even closer association with total health expenditure (THE) per capita (*r* = 0.870), which suggests that the available economic resources are less important than the overall priority given by governments to population health.

## What Is to Be Done?

If we assume that no population should be excluded from health research, what is to be done? The situation in the small island states is perhaps the easiest to address and, indeed, this is already happening through the expansion of academic consortia. Many already participate in a number of regional academic initiatives. These include the University of the South Pacific, with campuses in 11 countries, and the University of the West Indies, with campuses in Jamaica, Barbados, and Trinidad, as well as an Open Campus that reaches out to the remaining 13 participating countries and territories. The Caribbean states also benefit from the work of the Caribbean Epidemiology Center, based in Trinidad. Collaborations have been facilitated by enhanced transport links and especially the spread of the Internet. However, significant challenges remain. Internet connections remain poor in some Pacific Island states, such as Tonga, Vanuatu, and the Solomon Islands. The University of the South Pacific campus in Niue notes problems with “power supply, water, transportation, and absence of study facility" but that its “students are very motivated to succeed" [11]. These regional universities have embraced distance learning, often supported by part-time local tutors, and there is at least the basic infrastructure needed to undertake research.

Where there is a national research strategy, there may be regional and cultural specificities that pose barriers to adopting international scientific standards. One example of this problem occurs in the former Soviet countries. In 1928, Stalin introduced what was termed “Soviet science", with its adherents privileged over those using conventional scientific methods [12]. Soviet science was characterised by references to statements by the founding fathers of the major disciplines, as well Marx and Lenin, who were considered to have provided the basis for all subsequent discoveries. The most notorious example was the work of the agronomist Trofin Lysenko, whose rejection of Mendelian inheritance contributed to widespread failures of Soviet agriculture. In medicine, a regime that was unable to develop a modern pharmaceutical industry benefited from the rejection of concepts such as randomised controlled trials that would likely have revealed problems with the various electromagnetic and physical therapies being promoted in government hospitals [13]. The legacy of Soviet science persists, with research still being undertaken on, for example, the potential role of magnets to treat hypertension (based on the incorrect view that it is caused by increased blood viscosity) [14], and the use of many inappropriate mineral and vitamin preparations to treat a wide range of medical disorders [15],[16]. Here, the task is one of unlearning perceived wisdom. Such challenges create a different, and arguably more difficult, set than those that exist where there is no existing research support.

Another difficulty is in attracting and maintaining expertise in settings of low quantity and quality production of health science. Academic staff are aging, in part because those who would otherwise have retired remain dependent on income from teaching in the absence of adequate pensions, and also because they have been unable to recruit or retain talented younger staff. The career prospects in academia for young researchers, especially those who have trained in the West, are abysmal. Wages are several-fold lower than what they can obtain either in Western countries or in the private sector in their own countries. Domestic research funding is scarce and, in the post-Soviet countries, its distribution is determined by a gerontocracy whose ideas were shaped during the Soviet period, many of whom do not read English and, as a consequence, are unfamiliar with the international literature. Foreign qualifications are not recognised. Indeed, the authors are aware of how certain universities in some countries, such as Russia, disregard papers published in English, even when in leading international journals, in promotion processes. A few dedicated individuals continue to participate in research voluntarily, often in association with foreign collaborators, but without any domestic institutional support. However, their activities are inevitably highly constrained.

To continue with the post-Soviet example, we can draw lessons from glimmers of success in spite of the aforementioned issues. Russia has recently refocused its research strategy to support excellence and to recruit leading international scientists, especially but not exclusively Russians who have previously moved abroad [17]. Georgia, despite major financial constraints, has already made notable progress. It has embraced a policy of openness to international collaboration and has established foundations that offer skilled researchers, many trained abroad, a career structure. It has emphasised the importance of being able to read English. While accepting the limitations of the indicator used in this paper, it is noteworthy that it has achieved more than 50% more medical publications per 100,000 population than Russia (16.6 versus 11.2), and five times more than countries such as Ukraine (3.7) and Moldova (3.6). Indeed, Georgian public-health researchers are engaged in projects across the former Soviet Union [18].

The situation in Africa is challenging, but hopeful in parts. Many of the countries in Table 2 have suffered major conflicts in the past 15 years, including Niger, Liberia, Sierra Leone, Chad, Somalia, and Zimbabwe. Some are still considered fragile states, with weak governments struggling to deliver even the most basic protection for their populations. However, no African country appears in the top 60 when ranked by publications per 100,000 population. The six highest are Tunisia (72.1), Seychelles (57.8), South Africa (41.1), Gambia (38.5), Gabon (30.6), and the Republic of Congo (16.7).

**Figure 1:**
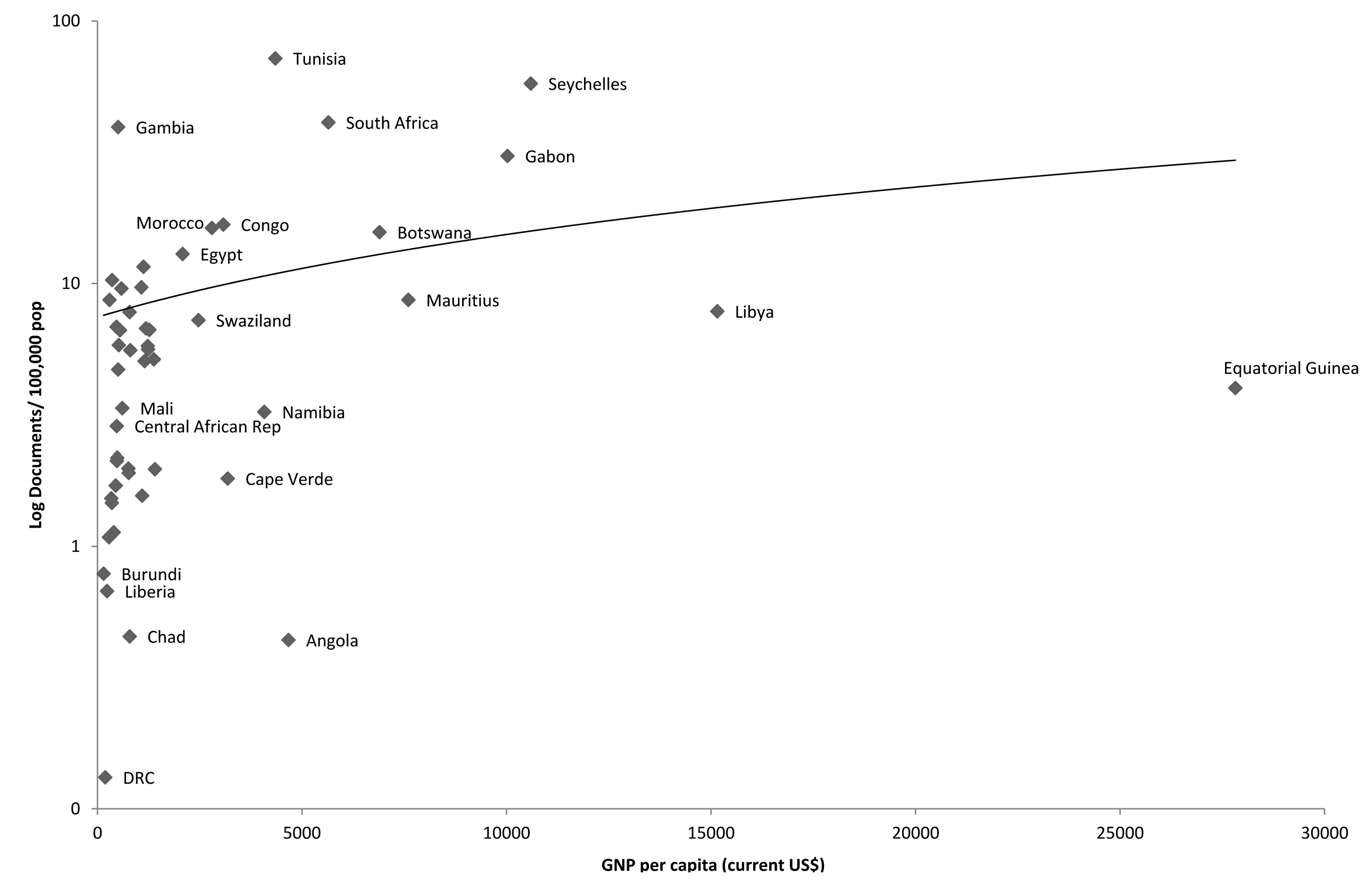
Association between publication output (1996–2010) and gross national product per capita (2008), Africa.

**Figure 2:**
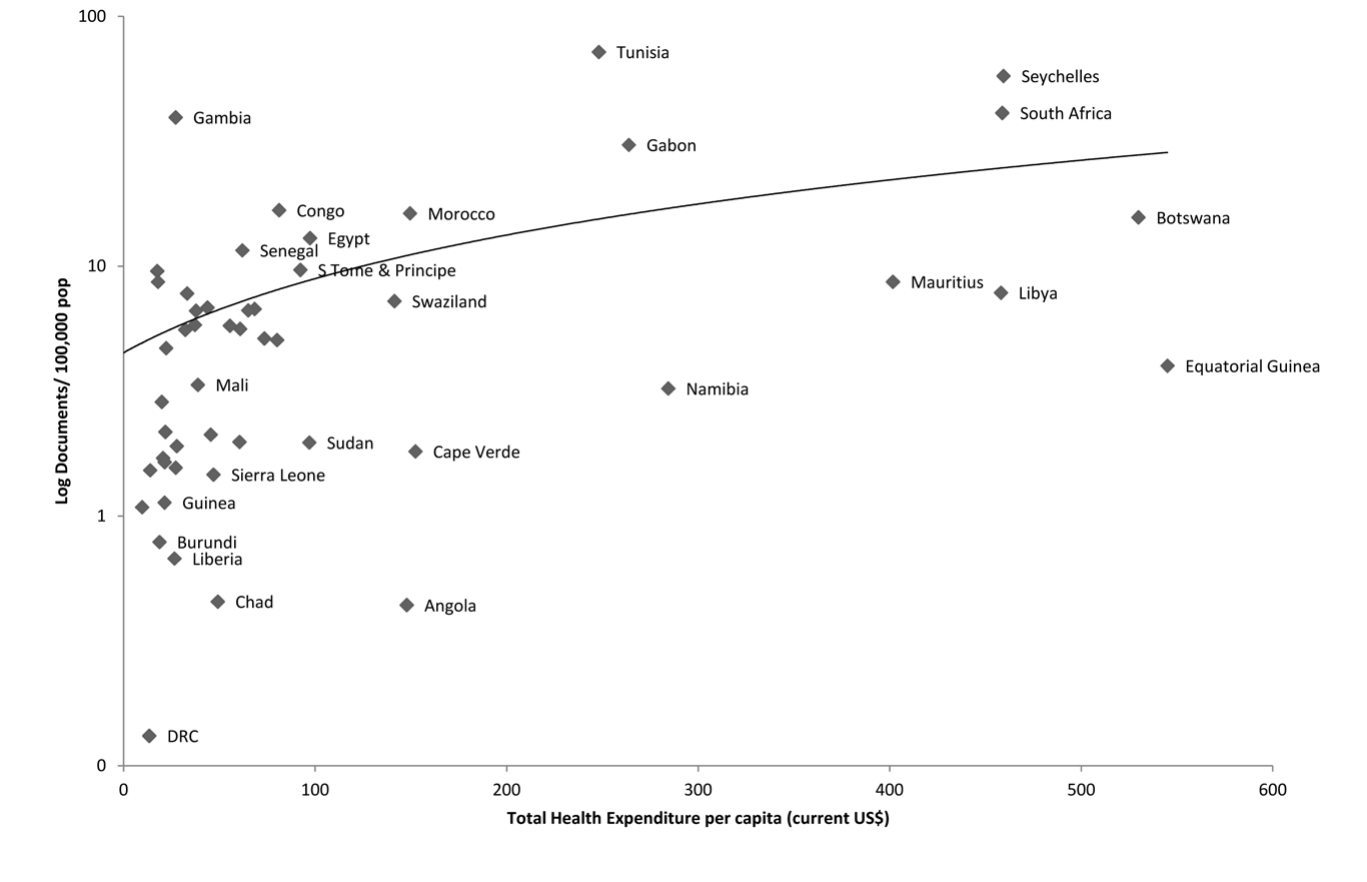
Association between publication output (1996–2010) and total health expenditure per capita (2008), Africa.

Some clues are apparent from a comparison of output with GNP and THE per capita (Figures 1 and 2). These confirm that having resources is not sufficient. Oil-rich and authoritarian, Equatorial Guinea and Libya both produce many fewer publications than would be expected. Seychelles and South Africa both have well-established higher education and medical sectors, albeit in the latter they have suffered from limited funding for many years. Yet, in contrast to the Seychelles, with which it otherwise has much in common, Mauritius does less well than expected. Tunisia has strong links with France, including the highly productive Institut Pasteur de Tunis [19]. Gambia hosts a major tropical medicine centre run by the United Kingdom Medical Research Council. The Medical Research Unit at the Albert Schweitzer Hospital in Gabon, another good performer, has a strong track record of project-based funding from leading international research funders and works closely with German researchers. The Republic of Congo also does better than would be expected and, in Brazzaville, the Congolese Foundation for Medical Research recently made a significant investment in capacity development and is collaborating actively with researchers in neighbouring countries. There are also a number of regional initiatives bringing together a number of African countries, such as the European Community-funded European and Developing Countries Clinical Trials Partnership, which includes 47 sub-Saharan African countries [20], and the South African National School of Public Health, which is training graduates from neighbouring countries [21].

It is, however, those countries with the least capacity that we are concerned with here. In addition to those in Table 2, there are a further six that have fewer than three publications per 100,000 population: Sudan, Lesotho, Rwanda, Madagascar, Algeria, and the Central African Republic. Many of these have previously been identified as lacking any capacity for postgraduate training for public health; in the same study, it was also noted how that measure of capacity in the Francophone and Lusophone countries was especially weak, a finding confirmed here [22]. This is likely to reflect the growing dominance of English in the international scientific literature [23], which will disadvantage those potential researchers unable to read it, a problem shared with the former Soviet countries.

All of these countries are on different trajectories and face different challenges. In each case the response will vary. For example, Rwanda, despite suffering up to a million deaths in the violence of 1994, is now making substantial progress in many sectors, including health care and higher education. This may be a country that might benefit from additional targeted investment in research capacity, building on the recently created National Center for Clinical Research, a development that has been supported by President Kagame [24]. However, further investment may be linked to progress in human rights [25]. On the other hand, the governments in Madagascar and Sudan have attracted international condemnation for failures of governance and human rights abuses, and effective responses may have to await resolution of these issues. Some other African countries either have no functioning central government, such as Somalia, or governments that have extremely limited capacity in any sector, such as Niger, Mauritania, and the Central African Republic. It is difficult to see what can be done in these countries without stability and significant strengthening of basic governance functions. Lesotho has the scope to strengthen its existing collaborations with South Africa, such as those with the Medical Research Council.

Then there are countries that are politically stable and have sufficient population and economic resources to support a research infrastructure, yet have so far failed to create one. These include Indonesia, Ethiopia, the Philippines (1.92 publications/100,000 population), and Algeria (2.28 publications/100,000 population). These countries may be able to learn from history. In the 1920s, the Rockefeller Foundation stimulated public health training and research by major grants to create academic centres in 21 countries, including the United Kingdom (the London School of Hygiene and Tropical Medicine), China, Yugoslavia, Canada, and Brazil. More recently, Atlantic Philanthropies have supported schools in Vietnam and South Africa, the Wellcome Trust has embarked on capacity-building programmes in India and Africa, and the Open Society Institute has supported public health programmes in central and eastern Europe [26]. Not all such developments have been funded by Western donors; the James P. Grant School of Public Health was established by the Bangladesh Rehabilitation Assistance Committee, the country's largest non-governmental organisation [27]. There is now a considerable body of experience with these initiatives. Key lessons learnt include the need for sustained investment (over a period of at least ten years), support for academic leadership and managerial skills and not just teaching and research skills, and the creation of career pathways for graduates [28].

Notwithstanding the current global financial crisis, there is an argument to be made for leading donors to explore the scope for strategic investment in higher education in some countries that have so far been neglected. Obvious emerging priorities are those countries of North Africa that are in the process of transitioning to democratic rule, and where research capacity has, so far, been very limited. For example, Ethiopia has made substantial achievements in health reform in the past two decades from a very difficult starting point, and there is now a high level of political commitment to investing in health [29].

Finally, there are a few countries in other parts of the world that, like some of those in Africa, are suffering from, or emerging from, the effects of extensive conflict. These include Afghanistan and Timor-Leste. In each, as in similar countries in Africa, the development of health research capacity will inevitably take second stage to the challenge of achieving peace, stability, and national reconstruction.

Throughout this review, we have noted the necessity of taking account of each country's specific circumstances. Nevertheless, at the risk of generalisation, we can identify four broad clusters of countries, defined according to their access to resources and commitment to building health research capacity, each of which may benefit from particular measures. These are summarised in Box 1.

**Box 1. Potential Opportunities for Investment in Building Capacity in Health Research**

We can tentatively identify four clusters of countries in which, although their national specificities differ, they may be amenable to a common set of strategies for promoting health research capacity.

*Cluster 1: Political commitment but limited health research capacity:* In a few countries there is evidence of a political commitment to health, but health research remains weak. In these countries a solution like an international health consortium could potentially have a substantial impact. Examples of this approach include the Fogarty International Research Collaboration, such as its International Tobacco and Health Research Program, and the Wellcome Trust International Collaborative Research Grant Scheme, which supports projects in the Pacific Islands. However, many donors and investors tend to focus on short-term results of research investments, favouring countries with existing research infrastructure and capacity. As a result, those countries which lack research infrastructure are often left behind (an illustration of the “inverse care law"). Through targeted investments, it may be possible to correct these international research imbalances. Investing in high commitment/low research countries may achieve a longer term, greater impact by stimulating a process within countries to produce and reward high-quality research. Examples include Botswana, Cape Verde, Swaziland, and Mauritius.

*Cluster 2: Political commitment and moderate health research capacity:* Several countries are regional leaders in producing research, typically those with greater economic resources and where a political priority is placed on health. Where such capacity exists, the focus of donor interaction may be to sustain and improve the quality of research, not just the quantity. Additionally, emphasis may be needed to reward and retain high-performing researchers within the country. Examples include South Africa and Seychelles. These countries may also be helped to support neighbouring countries or those facing similar challenges (e.g., Seychelles as a source of support for small island states).

*Cluster 3: Low political commitment and low research capacity:* In some countries where political commitment and research investment are both low, it is necessary to understand the reasons why. Where there is ongoing ethnic or military conflict, the emphasis may need to be on planning for the post-conflict period, for example by training researchers who may, at some point, return. In other cases, authoritarian political regimes may be opposed to research on health, particularly when evidence on adverse outcomes could be perceived as a political tool for dissent. There is little that can be done in such cases. However, there may be opportunities to support targeted research to raise awareness of health needs and identify interventions that could help raise the political priority given to health. A possible example might be Namibia.

*Cluster 4: Low political commitment and moderate health research capacity:* A few countries are producing significant amounts of research despite the absence of resources or, apparently, political commitment. These are countries hosting research institutions funded by and managed from high-income countries. They include Gabon, Gambia, and the Republic of Congo. These institutions are well placed to provide a focus for indigenous capacity development provided that donors support advanced training of local researchers.

## Conclusions

One speaker at the 2008 Global Ministerial Forum on Research for Health, in Bamako, Mali, in 2011 said “Countries don't need a national airline, but they do need a national health research strategy". Although there has been a steady increase in the participation by low- and middle-income countries in the international research community in recent decades, there are still many that lack anything resembling a health research strategy. The reasons vary. In some cases they are political, when regimes shun international engagement. In others they are geographical, as with small and often remote island communities. In others still they are historical, as in those countries that have emerged from conflict. In some, there may be little that can be done until there are governments in place that value the health of their populations and see the benefit of investing in the knowledge needed to address their problems. Yet, there are also a few where there is political commitment, and where relatively small investments in capacity could make a difference. Each must be considered individually. However, one thing is certain. None should be abandoned by the global health research community.
